# Trace Elements in Living Systems: From Beneficial to Toxic Effects

**DOI:** 10.1155/2017/8297814

**Published:** 2017-03-12

**Authors:** Marcin Mikulewicz, Katarzyna Chojnacka, Beata Kawala, Tomasz Gredes

**Affiliations:** ^1^Department of Dentofacial Orthopaedics and Orthodontics, Division of Facial Abnormalities, Medical University of Wrocław, Wrocław, Poland; ^2^Department of Advanced Material Technologies, Faculty of Chemistry, Wrocław University of Science and Technology, Wrocław, Poland; ^3^Department of Dentofacial Orthopaedics and Orthodontics, Medical University of Wrocław, Wrocław, Poland; ^4^Department of Orthodontics, Technische Universität Dresden, Dresden, Germany

There are two faces of trace elements: beneficial and toxic. Trace elements although present in trace quantities can play an essential role in living organisms; for instance, the ions are frequently bound to the active sites of enzymes. For different branches of science, trace elements definition is diverse, for example, in living organisms or in soil [1]. In some cases the trace elements cover at the same time microelements and toxic elements. The role depends primarily on dose or concentration.

Living organisms are exposed to trace elements from different sources, for example, diet, environment (water, soil, and air), or biomaterials. Depending on the route of exposure (e.g., oral, dermal, or inhalation), the effect related with a given dose would be different. Also chemical form is significant: for example, the fact that Cr(III) is micronutrient versus the fact that Cr(VI) is carcinogenic and mutagenic agent. This multiplicity of roles and effects causes large variability of different topics and issues related with trace elements. There are several techniques to evaluate the potential toxicity of trace elements in living systems: in vitro and in vivo, from laboratory elution tests, through molecular biology trials, animal studies, and ending with human usage tests.

On the other hand, trace elements can be also considered nutritional elements, micronutrients. They perform many important functions in living organisms. Deficiency of those nutrients is called “hidden hunger” [2]. For this reason, fertilizers, feed, and food products should supply the required dose of microelements. Particularly designed formulations are elaborated and new strategies of feed and food biofortification are being implemented [3].

The effect of trace metals on living organisms can be investigated directly by using various biomonitoring techniques, for example, hair mineral analysis [4, 5] or analysis of other noninvasive matrices (urine and saliva) [6]. In some cases using invasive matrices seems essential, for example, blood.

In this special issue there are papers that explore various concepts related with advantages and disadvantages of the presence of trace elements in living organisms. On one hand trace elements are nutritive; on the other hand in excessive dose they can pose toxic effects. There are several sources from which living organisms can be exposed to trace elements: diet, environment, and biomaterials [5]. As a result, it is important to assess the exposure to trace elements by in vitro and in vivo approach.

Therefore, in this special issue, the subjects discussed are widespread. Some papers concern trace elements in agriculture, some others concern exposure of living organisms from the environment, and other papers concern aspects related with biomaterials containing trace elements.

Biomaterials are widely used in medicine because of their desired properties. They have found a wide variety of applications: from hip prostheses, orthodontic appliances, prosthetic restorations, implants, and metallic plates (fracture repair) to surgical screws. Some of those materials are inserted for short period of time and others for a lifetime. Depending on the time of exposure and released doses, side effects related with insertion of a given biomaterial are different and determine its biocompatibility.

Biocompatibility is defined as the ability of a material to function in a specific application in the presence of an appropriate host response. Biocompatibility is assessed by appropriate test methods: in vitro and in vivo [7, 8]. In vitro methods are cost-effective and efficient [9] but do not fully reflect the real conditions in living systems. On the other hand, in vivo methods should be ethically accepted. For instance, determination of biosafety is important through assessment of the release of metal ions during orthodontic treatment [10].

It is known that there is no fully biocompatible material. Each alloy interacts with surrounding tissues, which may result in the release of trace element ions from metal alloys of biomaterials. An example of alloys used in biomaterials is orthodontic appliances.

Contemporary orthodontic treatment is based mainly on fixed orthodontic appliances. The main elements of those appliances (brackets, wires, and bands) are manufactured from metallic alloys such as stainless steel, nickel-titanium, and TMA (titanium-molybdenum alloy). Those alloys can be a source of exposure of metal ions, among others, Ni, Cr, and Cd, which have been proven to be mutagenic, cytotoxic, and allergenic [11]. Because a greater part of patients who undergo orthodontic treatment are children and teenagers previously mentioned issues are so important.

It has to be underlined that environment of oral cavity favors metal ion release from parts of orthodontic appliance. Saliva (pH), temperature, mechanical stress and/or damage, and bacterial colonization are all factors that initiate various types of corrosion processes (including deterioration) [12]. The other issue is the condition of enamel surface after debonding of the brackets/bands. Use of proper (advocated) technique and burs can reduce the surface damage.

The Guest Editors do hope that the present special issue would be interesting to investigators working in different branches of science related with trace elements and studying their both essential and nonessential or even toxic roles in living systems would find some useful information.

*Marcin Mikulewicz* *Katarzyna Chojnacka* *Beata Kawala* *Tomasz Gredes*

## References

[1] Kabata-Pendias A., Pendias H. (2001). *Trace Elements in Soils and Plants*.

[2] Ruel-Bergeron J. C., Stevens G. A., Sugimoto J. D. (2015). Global update and trends of hidden hunger, 1995–2011: the hidden hunger index. *PLOS ONE*.

[3] Subbaiah L. V., Prasad T. N., Krishna T. G., Sudhakar P., Reddy B. R., Pradeep T. (2016). Novel effects of nanoparticulate delivery of zinc on growth, productivity, and zinc biofortification in maize (*Zea mays* L.),. *Journal of Agricultural and Food Chemistry*.

[4] Dongarrà G., Lombardo M., Tamburo E., Varrica D., Cibella F., Cuttitta G. (2011). Concentration and reference interval of trace elements in human hair from students living in Palermo, Sicily (Italy). *Environmental Toxicology and Pharmacology*.

[5] Kempson I. M., Lombi E. (2011). Hair analysis as a biomonitor for toxicology, disease and health status. *Chemical Society Reviews*.

[6] Mikulewicz M., Kachniarz K., Chojnacka K. (2015). Exposure of cleft lip and palate patients to toxic elements released during orthodontic treatment in the study of non-invasive matrices. *PLOS ONE*.

[7] Schmalz G. (1997). Concepts in biocompatibility testing of dental restorative materials. *Clinical Oral Investigations*.

[8] Williams D. F. (2009). On the nature of biomaterials. *Biomaterials*.

[9] Wataha J. C. (2012). Predicting clinical biological responses to dental materials. *Dental Materials*.

[10] Chojnacka K., Mikulewicz M. (2014). Modelling of Cr and Ni ions release during orthodontic treatment:* in vitro* and *in vivo* methods. *Environmental Toxicology and Pharmacology*.

[11] Eliades T., Athanasiou A. E. (2002). In vivo aging of orthodontic alloys: implications for corrosion potential, nickel release, and biocompatibility. *Angle Orthodontist*.

[12] Mikulewicz M., Chojnacka K., Wołowiec P. (2014). Release of metal ions from fixed orthodontic appliance: an in vitro study in continuous flow system. *Angle Orthodontist*.

